# Optimization Of PVDF-TrFE Processing Conditions For The Fabrication Of Organic MEMS Resonators

**DOI:** 10.1038/srep19426

**Published:** 2016-01-21

**Authors:** Pierre-Henri Ducrot, Isabelle Dufour, Cédric Ayela

**Affiliations:** 1 Univ. Bordeaux, IMS, UMR 5218, F-33405 Talence, France; 2 CNRS, IMS, UMR 5218, F-33405 Talence, France; 3 Bordeaux INP, IMS, UMR 5218, F-33405 Talence, France

## Abstract

This paper reports a systematic optimization of processing conditions of PVDF-TrFE piezoelectric thin films, used as integrated transducers in organic MEMS resonators. Indeed, despite data on electromechanical properties of PVDF found in the literature, optimized processing conditions that lead to these properties remain only partially described. In this work, a rigorous optimization of parameters enabling state-of-the-art piezoelectric properties of PVDF-TrFE thin films has been performed via the evaluation of the actuation performance of MEMS resonators. Conditions such as annealing duration, poling field and poling duration have been optimized and repeatability of the process has been demonstrated.

Piezoelectricity is a convenient phenomenon used in MEMS (Micro-Electro-Mechanical Systems) because of its reversible effect, allowing the integrated actuation and detection of resonance with the same film. Despite the large success of inorganic piezoelectric materials such as PZT^1^ (Lead Zirconium Titanate), alternative materials, organic or not, are used to avoid the presence of heavy metals^2^. PVDF (Polyvinylidene fluoride) and its co-polymers, Parylene-C^3^ and Polyimide^4^ are bulk-polymers commonly used in MEMS. Piezoelectric composites, in which a polymer matrix contains ceramic particles, are also used because they combine the high coupling factors of ceramics and the mechanical flexibility of polymers^5^. Among the organic piezoelectric materials that are of primary interest due to their low cost and high flexibility, PVDF exhibits the best piezoelectric properties^6^. That is why it is the most commonly used piezoelectric polymer in electromechanical devices. Because of their attractive piezoelectric properties, PVDF and PVDF-TrFE (polyvinylidene fluoride-trifluoroethylene) can be found in numerous applications, including tactile sensors in the form of a flexible dome^7^ or as an active layer in MOS transistors^8^, pressure sensors^9^ or accelerometer sensors^10^ (where a mechanical stress induces charges in the piezoelectric layer), power harvesting applications^11^, and ultrasonic transducers^12^. PVDF is not used only for its piezoelectricity but also for its ferroelectricity for nonvolatile memories^13^ or waveguides^14^. A literature survey reveals that processing conditions of PVDF and associated copolymers vary from one work to another and, more importantly, there is no justification in the choice of these conditions.

The present work deals with the influence of several processing parameters on PVDF-TrFE piezoelectric properties. Indeed, PVDF is usually non-polar under its *α* phase. However, stretching it while applying a DC electric field conforms it into its dipolar *β* phase, which is of primary interest for piezoelectricity. The *β* phase can also be obtained directly by adding a co-polymer; in such a case, only electrical poling is necessary for piezoelectricity, i.e., the stretching is no longer necessary. Moreover, the crystallinity of PVDF-TrFE is better than that of PVDF, with a higher remanent polarization and a higher temperature stability (up to 100 °C)^6^,^15^. For this reason the co-polymer PVDF-TrFE has become popular in recent years and is thus chosen for study in the present work.

In order to study the influence of processing parameters on the piezoelectric properties of PVDF-TrFE, organic MEMS cantilever resonators are used in the present work. The micromachining method of our approach does not require the high-cost semiconductor manufacturing equipment used for silicon microfabrication and therefore drastically reduces the cost of the MEMS devices in comparison with those based on silicon technology^16^. We have demonstrated that actuation efficiency of the device is directly related to electromechanical properties (e.g., the *d*_31_ piezoelectric coefficient relating the longitudinal strain perpendicularly to the applied electric field) of PVDF-TrFE. This original approach is simple but efficient and accurate for a direct evaluation of the piezoelectric properties of processed PVDF-TrFE thin films. The architecture of the MEMS is shown in Fig. 1: it is composed of a PEN (Polyethylene naphthalate) substrate, in addition to bottom and top electrodes made of aluminum on both sides of the PVDF-TrFE layer. Typical dimensions of the cantilevers are 3.4 mm in length and 2 mm in width with an electrode width of 1 mm. Applying an AC electric field between the two electrodes makes the PVDF-TrFE layer shrink and stretch alternatively. The multi-layer effect leads to a flexural vibration of the cantilever. As shown in the following sections, the amplitude of the first mode of vibration serves as the basis in this work for evaluating the influence of the processing parameters on the value of the *d*_31_ coefficient.

## Results and Discussion

### Influence of poling

**Figure 2:**
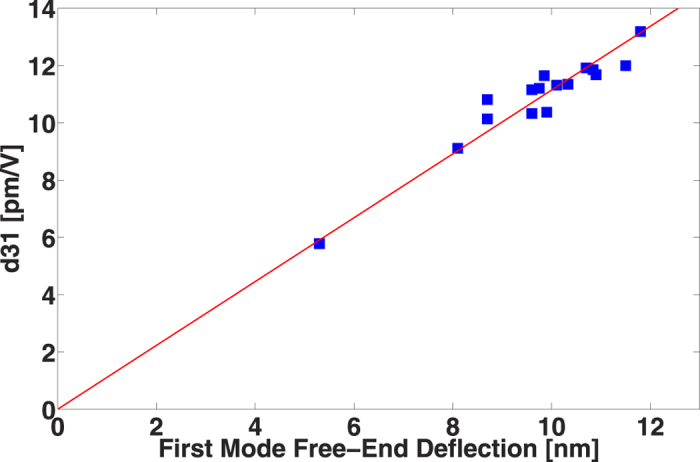
*d*_31_ coefficient versus first mode amplitude of the free-end deflection.

Using the equation (1) described in the methods part, *d*_31_ coefficients were calculated from measurements under quasi-static excitation and were then correlated to the amplitude of the first mode of resonance. As shown in Fig. 2, the direct proportionality between *d*_31_ and the amplitude of the first resonance mode is preserved. Theoretically, the first mode amplitude is *Q* (quality factor) times higher than the amplitude under quasi-static conditions, where *Q* is assumed to be independent of the *d*_31_ coefficient. Therefore, the results of Fig. 2 and the results from fundamental vibration theory are consistent, supporting the fact that a higher first mode amplitude corresponds to a higher *d*_31_ coefficient and validating the use of the first resonance mode amplitude as an evaluation criterion of the value of *d*_31_.

An important step in the process is the electrical poling of the PVDF-TrFE films. The goal of this step is to apply a strong electric field in order to align (at the nanoscale) all the dipoles with the electric field and, thus, to cause (at the macroscale) a significant piezoelectric response. Although values of the necessary electric field can be found in the literature, there is no clear justification regarding the choice of the poling conditions. For instance, an electric field of 70 V·μm^−1^ at 90 °C or 100 V·μm^−1^ at room temperature have been described in previous studies^7^,^17^ In fact, to our knowledge no rigorous study has been performed to justify these (or any other) values.

**Figure 3:**
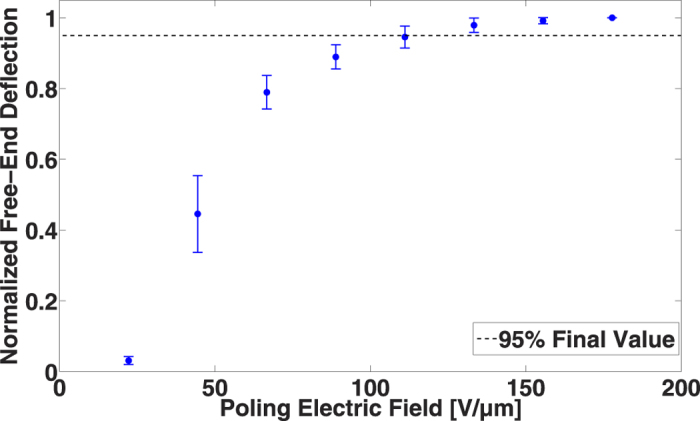
Influence of the electric field on the piezoelectric response of PVDF-TrFE over six devices.

To study accurately the influence of the electric field, all samples were annealed at 140 °C for 1 h after spin-coating and then poled for 30 min with a DC voltage. Figure 3 presents the results of the influence of the electric field on the piezoelectric response of PVDF-TrFE. The first mode amplitude is normalized in order to compare the results on six samples. According to this figure, if we consider 95% of the final measured value of deflection to be sufficient with a good repeatability, the necessary electric field is about 100 V·μm^−1^ under room-temperature conditions, thereby confirming experimentally the values specified in one of the earlier studies^17^.

**Figure 4:**
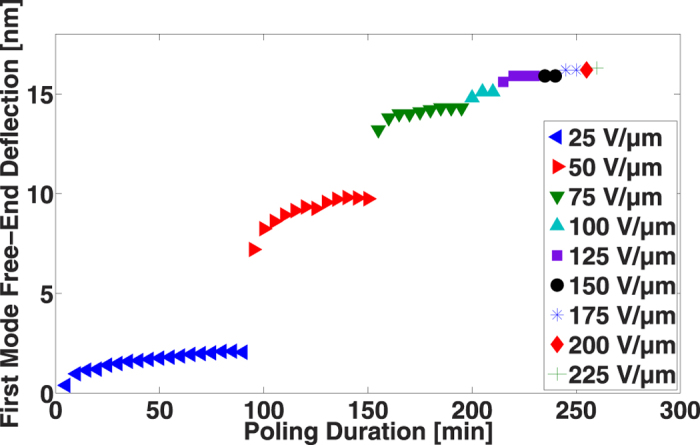
Effect of successive polarizations of PVDF-TrFE on the piezoelectric response.

To be sure that under 100 V·μm^−1^ a low piezoelectric response is due to a lack of electrical field and not related to the poling duration, one device has been poled sequentially over five-minute intervals at a fixed value of electric field, with response measurements being made after each of these poling intervals. After one set of these measurements, the process was repeated at an increased value of electric field. As seen in Fig. 4, if the poling field is too low, high values of *d*_31_ cannot be reached, even for long poling durations. More interestingly, again using 95% of the final value, we find again that the required strength of the electric field is 100 V·μm^−1^. This result clearly shows that the value of the electric field is much more important than the poling duration in determining the effectiveness of the poling process of PVDF-TrFE. Indeed, an incremental, step-by-step poling or a direct polarization at a high electric field leads to the same result.

**Figure 5:**
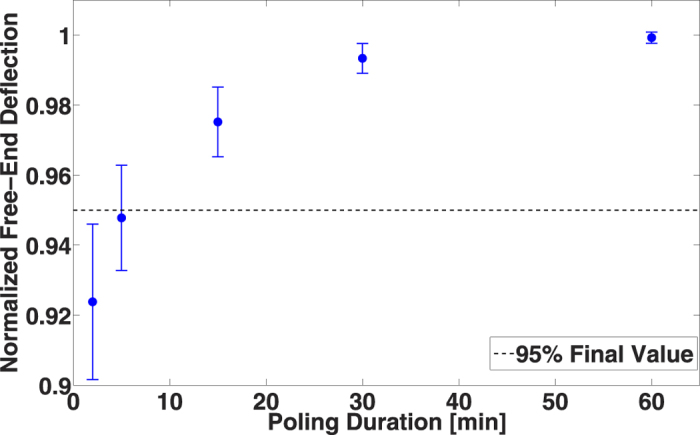
Influence of the poling duration at 100 V.*μ*m^−1^ over six devices.

As the poling field is an important parameter, the poling duration may play a role in piezoelectric properties. To confirm this influence, six samples have been poled at 100 V·μm^−1^ for different durations (Fig. 5). The results show that only five minutes at 100 V·μm^−1^ are sufficient to reach the maximum level of polarization of PVDF-TrFE with a standard deviation lower than 2%. This result is of particular interest since a short poling duration enables a rapid elaboration of piezoelectric thin films, an important criterion for the integration of PVDF-TrFE in industrial processes. Moreover, the influence of the poling duration on the piezoelectric property aging has been studied and can be seen in Supplementary Fig. S1. It results from this study that a long poling duration presents no advantages.

### Influence of annealing duration

**Figure 6:**
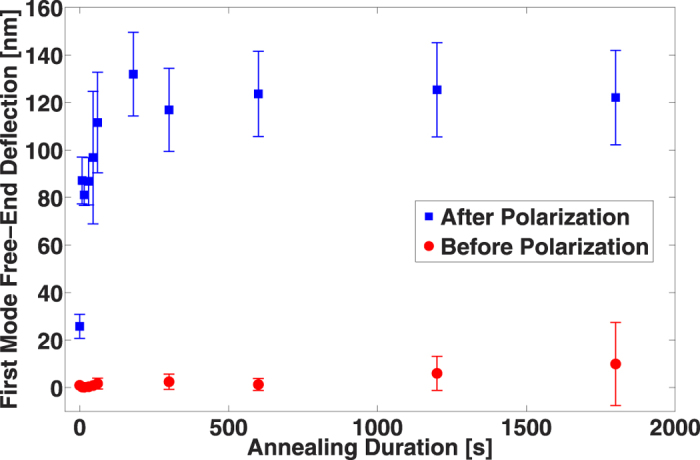
Influence of the annealing duration at 140 °C on the piezoelectric response with and without polarization, with an averaging over six devices for each measured data point.

As described in the fabrication section, PVDF-TrFE films are annealed after spin-coating. The influence of the annealing temperature has been already studied^18^,^19^. According to these two references, the optimal annealing temperature must lie between the Curie temperature (135 °C according to Piezotech) and the melting temperature (150 °C). Indeed, the paraelectric phase allows a better chain mobility, which leads to a higher crystallinity ratio (up to 91%). However, beyond the melting temperature, the crystallinity ratio decreases. It is also demonstrated that the better the crystallinity is, the higher the remanent polarization is. That is why the temperature of 140 °C has been chosen. Although the influence of the annealing temperature is well known, the influence of the annealing duration is not reported. Thus, samples were prepared and annealed at 140 °C (or not) for different durations, while measurements of the first mode amplitude, with an applied AC voltage of 210 mv, with and without a polarization step for 5 min at 100 V·μm^−1^, have been performed (Fig. 6). It can be seen that, before the poling step, samples present a residual polarization even after being annealed beyond the Curie temperature. Nevertheless, after polarization the piezoelectric response increases with annealing duration and the amplitude is much higher than without polarization, highlighting the importance of the poling step. Moreover, the results show that only a few minutes of annealing are necessary to achieve the best piezoelectric property of PVDF-TrFE, in contrast to times on the order of hours that can be found in the literature^18^,^19^. This result suggests that a large mobility of PVDF-TrFE chains occurs during annealing. In the case of polarization, the increase of the piezoelectric response versus the annealing duration indicates a better crystallinity for samples that have been sufficiently annealed (>3 min). The standard deviation is quite high, which may be explained by the fact that the position of the two electrodes is very important and is unique for each device. As for the poling duration, the influence of the annealing duration on the piezoelectric property aging has been studied and the results are shown in Supplementary Fig. S2. The same conclusion as for the poling duration can be made: a long annealing duration presents no advantages.

### Application to integrated organic resonators

**Figure 7:**
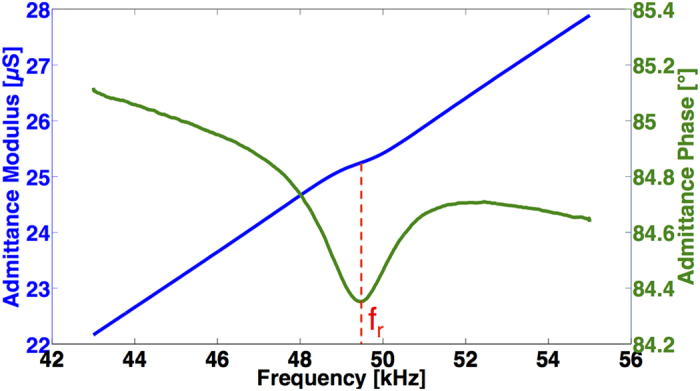
Admittance modulus and phase measurement centered on a resonance frequency.

The optimization of PVDF-TrFE processing conditions has been studied through actuation performance of organic cantilevers in order to have state-of-the-art values of the *d*_31_ coefficient, which is of main interest for such structures. However, thanks to the direct piezoelectric effect, it is also possible to detect electrically the motion of the cantilevers. Indeed, due to the mechanical strain in the cantilever, the PVDF-TrFE layer generates charges that can be monitored by impedance analysis. Figure 7 presents the admittance (modulus and phase) of a resonating cantilever measured by an impedance analysis in air. There are 1600 measured data points for one spectrum and each measured point is averaged fifty times. The resonance captured in the admittance measurement corresponds to a higher mode than the first one that is a combination of flexion and torsion (it has been checked with the vibrometer). In fact, the electric response is proportional to the vibration velocity and it appears that this mode presents the highest velocity. That is why this mode has been chosen to illustrate the electrical detection of the motion of the cantilever. Because of the geometrical capacitance due to the architecture of the structures (two electrodes separated by a dielectric material), the spectrum of the admittance modulus and phase does not correspond to a classical spectrum (with a peak for the modulus and a leap of 180° for the phase). Nevertheless, it is possible to separate the motional part from the coupling (with a post-measurement treatment) and to determine the electrical model of the resonator (a RLC branch in parallel to a capacitance *C*_0_ and a resistance *R*_0_). The identification of the terms gives the following orders of magnitude: *R* = 4.5 MΩ, *C* = 30 f F, *L*=350 H, *C*_0_ = 80 pF and *R*_0_ = 450 kΩ. Therefore, with the optimized processing conditions of PVDF-TrFE determined in the present work, organic resonators with integrated actuation and detection can be easily realized and at low cost.

## Conclusions

This work has demonstrated, using a rigorous approach, the optimization of processing conditions for the fabrication of piezoelectric thin films using the co-polymer PVDF-TrFE. Indeed, it has been proven that the necessary poling electric field at room temperature is 100 V·μm^−1^; below this value, PVDF-TrFE is not well-poled and beyond this level, there is no clear improvement in the piezoelectric properties. Also shown is that the necessary poling duration to achieve a sufficiently strong electric field is very short (≈5 min). It has also been demonstrated that the necessary annealing duration at 140 °C for 4 μm thin films is also quite short (≈3 min). A state-of-the-art average value of 11 pm·V^−1^ for the *d*_31_ coefficient was estimated over 16 structures after stabilization of the piezoelectric properties. The results of the study indicate that optimization of the process/fabrication parameters can result in organic resonators with integrated actuation and detection that may be fabricated in less than one day, which is extremely attractive from an industrial-use perspective.

## Methods

### Fabrication of devices

**Figure 8:**
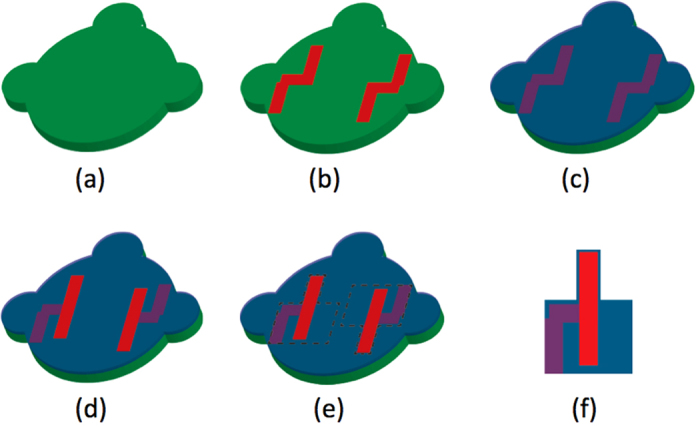
Processing steps of the cantilevers: (**a**) PEN substrate, (**b**) bottom electrode patterning, (**c**) PVDF-TrFE spin-coating and annealing, (**d**) top electrode patterning, (**e**) cutting of the cantilever shape, (**f**) final structure.

Here, a rapid and extremely low-cost fabrication process has been developed for the fabrication of organic MEMS. It starts with a 50 μm thick PEN film, used as a substrate. The desired shape of the substrate, as shown in Fig. 8, is obtained by means of a vinyl cutting machine Craft RoboPro CE6000 (Graftec Craft ROBO Pro) and is cleaned with isopropanol. Subsequently, 30 nm of aluminum is evaporated through a PET (Polyethylene terephthalate) shadow mask to pattern the bottom electrode. This is followed by the deposition of the PVDF-TrFE layer. A powder of PVDF-TrFE (75–25% in mole), provided by Piezotech, is dissolved in 2-butanone with a mass content of 20%. This solution is then spin-coated at 3500 rpm for 45 s with a ramp of 1 s, giving a thickness of about 4 μm. Two annealing steps are then performed: first, at 50 °C for 10 min to evaporate the solvent, followed by a 140 °C period of varying durations (one of the parameters to be studied) to improve crystallinity. The top aluminum electrode is then evaporated under the same conditions as the bottom one through a PET shadow mask. To finish the process, the shape of the cantilever is obtained simply by cutting the PEN substrate with the piezoelectric material using the cutting machine before gluing the resulting device onto a glass blade with double-sided adhesive tape, leaving the cantilever freely suspended. Finally, the PVDF-TrFE layer is poled with a DC electric field applied by connecting robotized electrical probers (provided by Imina Technologies) to the electrodes. The voltage and poling duration are the two parameters of primary interest in the present study.

### Determination of d_31_ coefficient

As mentioned previously, the criterion to evaluate the influence of processing parameters on the piezoelectric properties of PVDF-TrFE is the amplitude of the first out-of-plane mode of resonance because it is an easy and a convenient characteristic to be measured. The dynamic behavior of the piezoelectric organic MEMS resonators is obtained using a laser Doppler vibrometer MSA-500 from Polytec. The amplitude of the first out-of-plane flexural mode is measured at the free-end of the cantilevers, where the amplitude is maximal. The value of the applied voltage for the actuation for all measurements, with some indicated exceptions, is 21 mV. The important piezoelectric property that must be determined in our case is the *d*_31_ coefficient. For this reason we must establish a model relating the value of this coefficient to the tip displacement amplitude as measured by the vibrometer. Such a model exists^20^ for cantilevers composed of two layers:


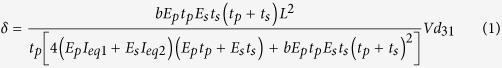


with *δ* the free-end deflection, *V* the applied voltage, *E*_*s*_ and *E*_*p*_ the Young’s moduli of the substrate and the piezoelectric layer, respectively, *t*_s_ and *t*_*p*_ the thicknesses of the substrate and the piezoelectric layer, respectively, *L* the cantilever length, *b* the width, *I*_*eq*1_ and *I*_*eq*2_ the equivalent second moment of area of the piezoelectric part and substrate part, respectively. This equation is only valid for a quasi-static actuation. The piezoelectric cantilevers are thus actuated at low frequency (200 Hz), far from resonance (≈1.8 Hz). However, if we take into account the electrodes, the cantilever is composed of four layers whereas the model considers only two layers. For this reason the bottom electrode and the substrate are merged together as are the top electrode and the PVDF-TrFE layer. The calculation of the equivalent second moment of area for the latter becomes


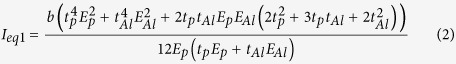


with *E*_*Al*_ and *t*_*Al*_ being the Young’s modulus and the thickness of the top (aluminum) electrode. In an analogous manner, *I*_*eq*2_ is calculated for the substrate and the bottom electrode.

## Additional Information

**How to cite this article**: Ducrot, P.-H. *et al*. Optimization Of PVDF-TrFE Processing Conditions For The Fabrication Of Organic MEMS Resonators. *Sci. Rep*. **6**, 19426; doi: 10.1038/srep19426 (2016).

## Supplementary Material

Supplementary Information

## Figures and Tables

**Figure 1:**
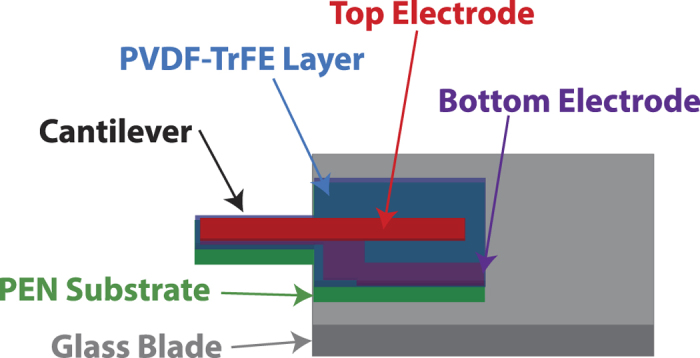
Architecture of the organic cantilevers used in this work.
