# Supplementary material for: Optimization Of PVDF-TrFE Processing Conditions For The Fabrication Of Organic MEMS Resonators
Source: Sci Rep. 2016 Jan 21;6:19426. doi: 10.1038/srep19426 (PMC4726305; doi:10.1038/srep19426)
Supplement: Supplementary Information [file srep19426-s1.pdf]

OPTIMIZATION OF PVDF-TRFE PROCESSING CONDITIONS FOR THE FABRICATION OF ORGANIC MEMS RESONATORS

Pierre-Henri Ducrot, Isabelle Dufour, Cédric Ayela

Supplementary information

Figure S1 presents the piezoelectric property aging for samples that have been poled at 100 V.μm^{-1} for 2 min, 5 min, 15 min and 30 min. A rapid decrease in the first mode free-end deflection, which occurs in the first hours, is observed followed by a progressively smaller decrease over time. There are no notable differences between the samples. Thus, this study shows that a long poling duration presents no advantages.

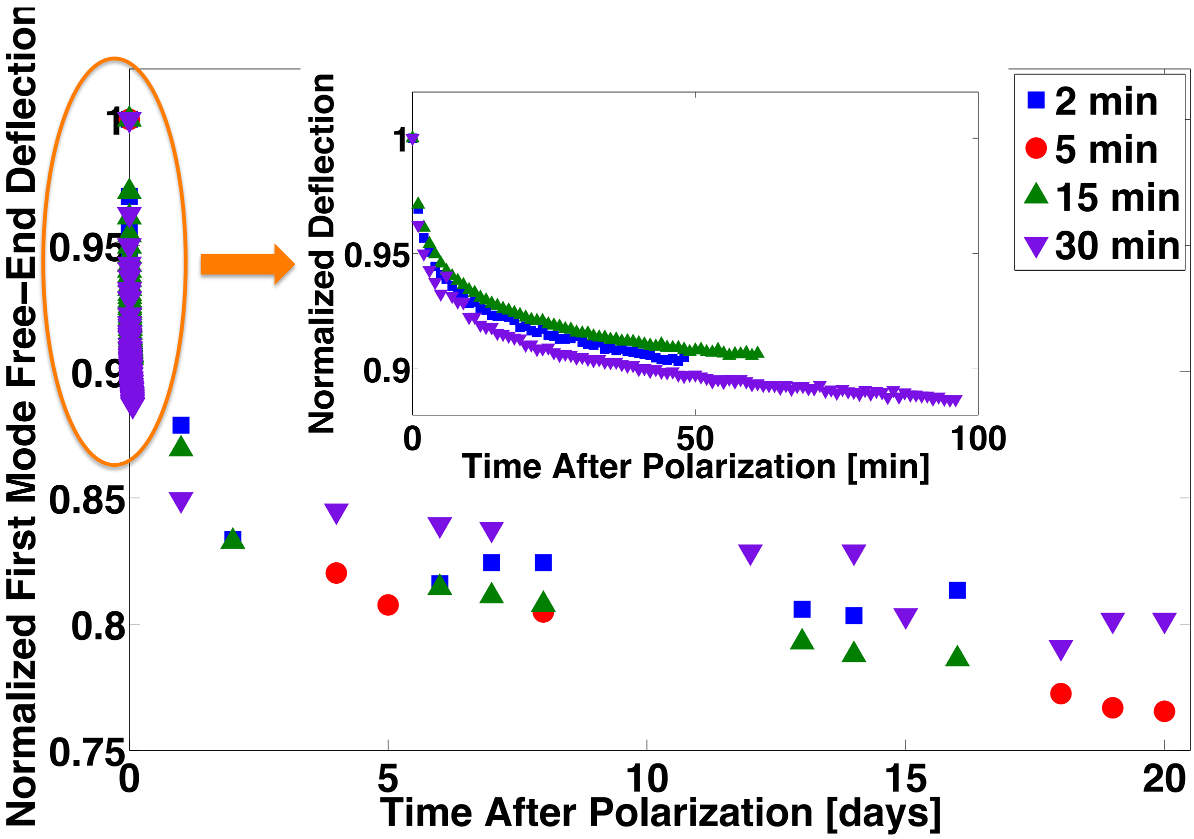

Figure S1. Piezoelectric property aging for three different poling durations: 2 min, 5 min, 15 min and 30 min.

As for the poling duration, it is interesting to explore the impact that the annealing duration has on the piezoelectric aging with samples that have been annealed à 140 °C for 3 min, 10 min, 20 min and 30 min (Fig. S2). Once again, a large decrease in d_{31} occurs in the first hours. The sample that has been annealed for 30 min seems more stable but the one that has been poled for 20 min presents the largest decrease in piezoelectric properties. Therefore, we conclude that another mechanism must be responsible for the decrease in the piezoelectric response, one which is independent of annealing and poling durations.

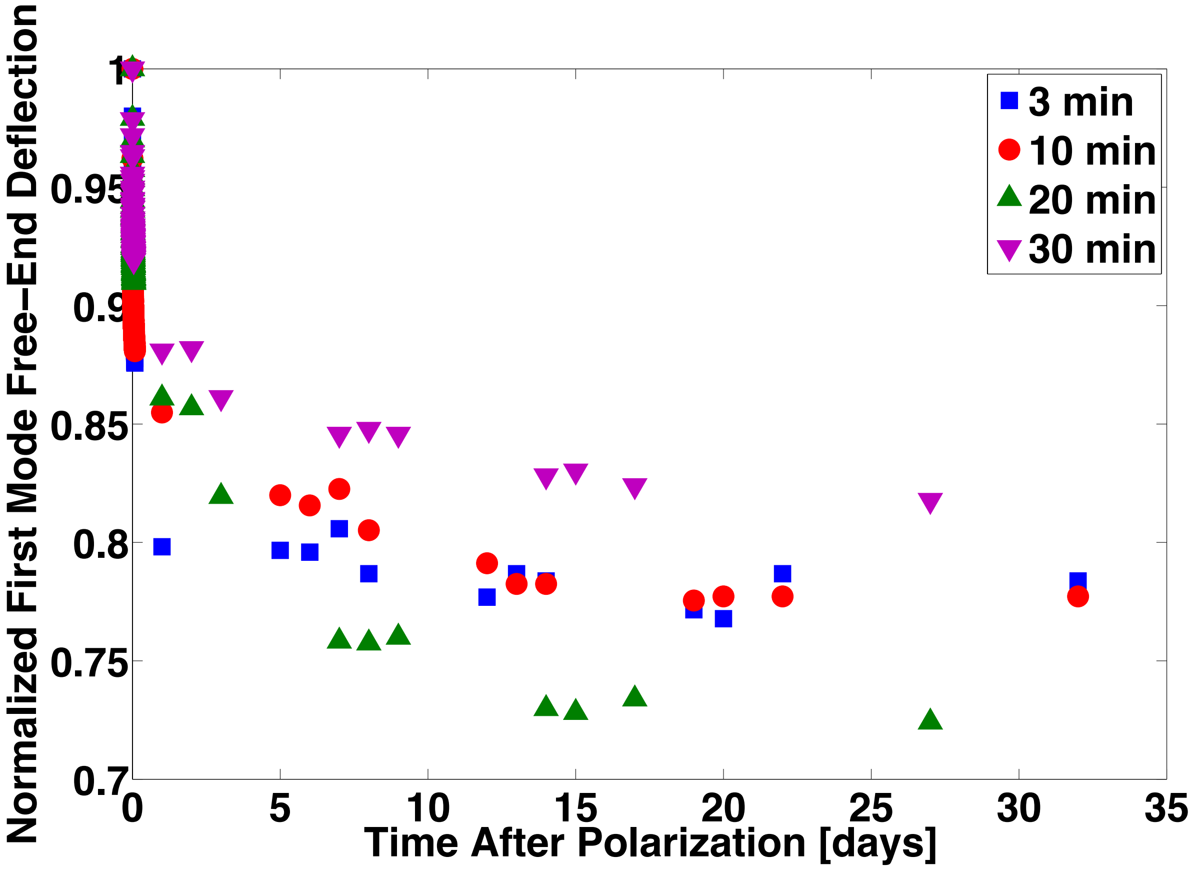

Figure S2. Piezoelectric properties ageing for four different annealing durations: 3 min, 10 min, 20 min and 30 min.
